# Comparative Effects of Mulligan’s Mobilization, Spinal Manipulation, and Conventional Massage Therapy in Cervicogenic Headache—A Prospective, Randomized, Controlled Trial

**DOI:** 10.3390/healthcare11010107

**Published:** 2022-12-29

**Authors:** Gopal Nambi, Mshari Alghadier, Elturabi Elsayed Ebrahim, Arul Vellaiyan, Jaya Shanker Tedla, Ravi Shankar Reddy, Venkata Nagaraj Kakaraparthi, Osama R. Aldhafian, Naif N. Alshahrani, Ayman K. Saleh

**Affiliations:** 1 Department of Health and Rehabilitation Sciences, College of Applied Medical Sciences, Prince Sattam bin Abdulaziz University, Al-Kharj 16278, Saudi Arabia; 2 Department of Nursing, College of Applied Medical Sciences, Prince Sattam bin Abdulaziz University, Al-Kharj 16278, Saudi Arabia; 3 Department of Medical Rehabilitation Sciences, College of Applied Medical Sciences, King Khalid University, Abha 62529, Saudi Arabia; 4 Department of Surgery, College of Medicine, Prince Sattam bin Abdulaziz University, Al-Kharj 16278, Saudi Arabia; 5 Orthopedic Surgery Department, King Fahad Medical City, Ministry of Health, Riyadh 12231, Saudi Arabia; 6 Department of Orthopedic, Faculty of Medicine for Girls, Al-Azhar University, Cairo 4434003, Egypt

**Keywords:** cervicogenic headache, Mulligan mobilization, spinal manipulation, massage therapy

## Abstract

Background: There is ample evidence supporting the use of manual therapy techniques for the treatment of cervicogenic headache (CGH). Objective: The objective of this study was to find and compare the effects of different manual therapy approaches to cervicogenic headache. Methods: A randomized, controlled study was conducted on 84 CGH participants at the university hospital. The participants were divided into a Mulligan mobilization therapy group (MMT; n = 28), a spinal manipulation therapy group (SMT; n = 28), and a control group (Control; n = 28); they received the respective treatments for four weeks. The primary outcome (CGH frequency) and secondary outcomes (CGH pain intensity, CGH disability, neck pain frequency, pain intensity, pain threshold, flexion rotation (right and left), neck disability index, and quality of life scores) were measured at baseline, after 4 weeks, after 8 weeks, and at a 6-month follow-up. The one-way ANOVA test and repeated measures analysis of variance (rANOVA) test were performed to find the difference between the inter- and intra-treatment group effects. Results: Four weeks following training, the MMT group showed a statistically significant difference in the primary (CGH frequency) and secondary (CGH pain intensity, CGH disability, neck pain frequency, neck pain intensity, flexion rotation test, neck disability index, and quality of life) scores than those of the SMT and control groups (*p* < 0.001). The same difference was seen in the above variables at 8 weeks and at the 6-month follow-up. At the same time, the neck pain threshold level did not show any difference at the 4-week and the 8-week follow-up (*p* ≥ 0.05) but showed statistical difference at the 6-month follow-up. Conclusion: The study concluded that Mulligan’s mobilization therapy provided better outcomes in cervicogenic headache than those of spinal manipulation therapy and conventional massage therapy.

## 1. Introduction

A headache is the most common symptom that usually affects 90% of the world population. A total of 66% of men and 57% of women experience this type of pain at least once a year [1]. A cervicogenic headache (CGH) is one type of headache, and it occurs commonly due to neck dysfunctions and the pain is usually referred from the neck region. The positive association between the neck dysfunction and CGH is found in many studies. The prevalence of this problem is more common in women than men [2]. It is also called a secondary headache and grossly affects the common population, putting considerable burden on the public health and having an economic impact and pressure on the community [3]. The prevalence rate of CGH varies from 4.6% to 18%, and it varies according to different factors [4]. It was observed that 30–60% of the world’s population has experienced this type of pain and has spent huge medical expenses on it [5]. It was also noted that CGH accounted for 157 million days of work absenteeism in the USA each year, which resulted in a loss of about 50 billion US dollars each year [6].

The diagnosis of cervicogenic headache is confirmed on the basis of subjective information and by performing a physical examination [7]. Pain in the neck region, reduced cervical range of motion (ROM), and loss of muscle property are usually noted during physical examination [8]. The flexion rotation test (FRT) is a diagnostic tool for CGH, and it is a reliable and valid tool for measuring upper cervical motion [9,10]. It was observed that medical and pharmacological management for cervicogenic headache has harmful side effects [11]. Additionally, there is a lack of safe, effective, and cost-effective physical therapy treatment for cervicogenic headache patients. There are a wide variety of holistic and integrative approaches available in the treatment of CGH, such as patient education, ergonomic guidance, positional therapy, and strengthening exercises [12]. Nevertheless, 34% of Americans receive physical therapy treatment in the form of soft tissue therapy and manual therapy for neck pain and CGH [13].

Generally, manual therapy techniques in physical therapy for a cervicogenic headache are joint mobilization and manipulation techniques [14]. The Mulligan Mobilization Technique (MMT) is a common joint mobilization technique, in which the patient performs active movement while the therapist glides the concerned joint [15]. In manipulation techniques, spinal manipulative therapy (SMT) is commonly used for neck-related pain and cervicogenic headache. Recent studies and reviews support the wide application of SMT in CGH and have shown its superior effects [16,17,18] over other treatments. Massage therapy is a treatment conventionally used by many physical therapists and chiropractors; it can also help to improve the symptoms in patients with cervicogenic headache.

There is a large amount of evidence supporting the use of manual and manipulative therapy techniques for CGH. However, there is a lack of information comparing the efficacy of manual and manipulative therapy techniques in cervicogenic headache, especially with respect to its clinical and functional aspects. Furthermore, the limitations noted in the previously published articles were lack of comparison in manual therapy approaches, poor study methodology, and low sample size trials. To date, no study has resolved these limitations related to the management of CGH. Additionally, there were differences in patient-centered outcomes between Mulligan mobilization, spinal manipulation, and conventional physical therapy for the care of CGH up to six months. Therefore, the aim of our study was to compare the effects of Mulligan mobilization, spinal manipulation, and conventional massage therapy in the management of cervicogenic headache patients. The study hypothesis was as follows: Mulligan mobilization is more effective than spinal manipulation and conventional massage therapy in cervicogenic headache patients.

## 2. Materials and Methods

### 2.1. Trial Design

This study was a prospective, single-blinded, parallel-group, randomized, controlled study. The study was initiated in June 2020, and the required participants were screened and recruited from University Hospital, Al Kharj, Saudi Arabia by a general physician with 20 years of experience, as per the headache classification guidelines defined by the International Classification of Headache Disorders—ICHD 3 [19]. The participants were randomly assigned to the Mulligan mobilization therapy (MMT; n = 28) group, the spinal manipulation therapy (SMT; n = 28) group, or the control (Control; n = 28) group. The study was designed as per the regulations of the CONSORT guidelines, and no notable changes were done during the execution of the study. Ethical approval was obtained from the Department Ethical Committee (DEC), with a reference number of RHPT/020/27, dated 15/03/2020. The study protocol and the informed consent forms were approved by the DEC. The trial was executed in accordance with the ethical guidelines laid down by the 1964 Declaration of Helsinki and was registered prospectively in the clinical trial registry (http://ctri.nic.in), with the registration number CTRI/2020/06/025600.

### 2.2. Participants

Participants with a history of chronic CGH (>3 months) and in the age group of 18 years to 60 years were allowed to participate in the study.

#### 2.2.1. Inclusion Criteria

- CGH pain intensity between 3 and 8 on 10-point pain scale;
- CGH due to cervical spine dysfunction;
- Reduced cervical motion;
- Neck pain followed by headache;
- Patients with neck stiffness and movement restriction were included.

#### 2.2.2. Exclusion Criteria

- Participants with other types of headache (migraine);
- Headache due to other causes (sinus, tumor, neural, or temporomandibular joint issues), and any type of physical therapy treatment in the past three months;
- Any contraindications to manual and manipulative therapy (fracture, instability, osteoporosis, arthropathy, or neural symptoms);
- Using analgesics or corticosteroids;
- Metastasis;
- Cardiac conditions (stroke, hypertension, or syncope);
- Neurological conditions (radiculopathy, myelopathy, or disc problems);
- Spinal cord problem;
- Previous brain and spinal cord surgery patients were excluded.

The eligible participants were recruited from King Khalid Hospital and University Hospital, Al Kharj, Saudi Arabia. The recommended study protocols were provided, and the study was executed at the Department of Physical Therapy and Health Rehabilitation, Prince Sattam bin Abdulaziz University, Al Kharj, Saudi Arabia.

### 2.3. Interventions

The approved interventions were provided by licensed physical therapists with 15–20 years of clinical experience in manual and manipulation therapy. All the participants of the study were compensated with free physical therapy treatment. First, they underwent 5 min of hydrocollator hot pack treatment on the neck and upper back region to relax the muscles. Next, the participants’ cervical vertebral joints and neck muscles were examined to identify the joint dysfunction. The respective mobilization approaches were provided to the participants as per the study protocol. Intervention bias was reduced by using fixed treatment procedures, and standard forms were used to document the details of treatment delivery and its follow-up. All the participants were instructed not to take any other forms of treatment during the study period. The treatment was given 4 times a week for 4 weeks to all the groups.

#### 2.3.1. Mulligan Mobilization Therapy (MMT)

The first group received Mulligan’s sustained natural apophyseal glide (SNAG) as the treatment of choice [16]. Each patient was asked to sit comfortably, and the treating therapist stood beside the patient. The patient’s head was free and cradled between the therapist’s right forearm and body, and the therapist stood at the patient’s right side. The therapist then placed his right index, middle, and ring fingers at the base of the occiput and kept his right little finger over the spinous process of C2. Next, with the lateral border of his left thenar eminence, gentle pressure was applied in a ventral and upward direction (45 degrees) over the right little finger. The contacting hand was relaxed, and the source of the power of gliding was delivered from the left hand. At the same time, pressure was applied by the left index finger to move the lower vertebra forward, and the slack was taken up. Gliding was applied rhythmically (three times per second) ten times, and the width of the gliding started from the middle to the end. The therapist continuously glided the joint and asked the patient to actively move their head towards the side of dysfunction and pain. When the patient moved their head, the therapist glided the spinous process ventrally and maintained the SNAG technique for 10 s. This technique was repeated 10 times for about 8 min. At the end of the mobilization, the patient was asked to passively rotate their neck and over press at the end range. 

#### 2.3.2. Spinal Manipulation Therapy (SMT)

Spinal manipulation therapy (SMT) is a high-velocity low-amplitude thrust (HVLAT) maneuver, defined by Peterson and Bergman [20]. This technique was provided by a trained manipulative therapist after assessing each participant at each visit through physical examination and proper palpation techniques. He identified and documented the sites of dysfunction in the cervical region and manipulated the region based on the study recommendations. SMT was not continued if the participant elicited any new contraindications or in the absence of symptoms, such as absence of pain or joint dysfunction. The participant was asked to lie comfortably in a supine position, with arms and legs inside and neck in neutral position. The therapist stood at the head of the patient, and the patient’s head was held like a cradle. One hand of the therapist was over the chin of the patient, the other hand was placed on the posterior aspect of the occiput, and the manipulation was performed in right and left directions. The movement was first away from the pain, and then the movement was towards the pain. SMT is a bimanual technique where the pre-manipulation rotation of 30°–45° is done away from the side of pain and manipulation is done towards the side of pain, with high velocity and low amplitude thrust. The range of rotation depends upon the level of the target vertebrae.

#### 2.3.3. Conventional Massage Therapy (CMT)

In this group, the participants received conventional massage therapy for 15 min using a Himalaya massage cream—Gold, New Delhi, India. The patient was asked to lie in a supine position, and the external occipital protuberance of the occiput was rested on a folded towel. The therapist stood at the head of the patient, and circular kneading was given on both sides of the cervical vertebra from C7 to C1 using the tip of the middle finger. This maneuver was performed three times for each cervical vertebra, from distal to proximal. Next, the head was rotated to the opposite side, and the same maneuver was performed on the levator scapula, sternocleidomastoid, scalene, and upper trapezius muscles from insertion to origin. This technique was also performed on the contralateral side and was repeated three times. Finally, the same circular kneading was applied over the longissimus capitis, splenius capitis, semispinalis capitis, and suboccipital muscles in the centripetal direction and was repeated three times [21].

All the participants in three groups were taught and instructed to do the neck isometric exercises daily, 3 times each day. In these exercises, the participant kept their palm over their forehead and resisted forward neck movement; this resistance was held for 10 s and repeated 15 times. Similarly, resistance was given on the back and both sides of the head, and the same maneuver was repeated. The participants were instructed to continue these exercises even after four weeks and also instructed strictly not to share the treatment information with fellow participants. The problems, questions, and concerns related to the study were collected after every session from all the participants and solved by the treating therapist. The interventions (Mulligan mobilization, spinal manipulation, and conventional massage therapy) were given by three different therapists, and they were aware of the research objectives. The potential benefits, harms, and discomforts of the treatment protocol were informed to the study participants through an informed consent form.

### 2.4. Outcomes 

The primary and secondary outcomes were measured by a blinded therapist in the department at each time point, such as at baseline, after 4 weeks, after 8 weeks, and at a 6-month follow-up.

#### 2.4.1. Primary Outcome

CGH frequency: This was a self-reported measure, in which the patients were asked to report the number of CGH days in 4 weeks through a medical log book, which was recorded every day at night [22].

#### 2.4.2. Secondary Outcome

CGH pain: The intensity of CGH pain was measured on a 10-point visual analogue scale (VAS). Patients were asked to mention the intensity of pain on a 10 cm scale, in which 0 denoted ‘no pain’ and 10 denoted ‘maximum intolerable pain.’ This is a valid and reliable (ICC = 0.60–0.77) tool for measuring CGH pain intensity and was evaluated at baseline and follow-up time points [23]. 

CGH disability: A 6-item Headache Impact Test (HIT) was used to measure the disability status of CGH patients, and it is a valid and reliable (ICC = 0.83–0.87) tool for measuring CGH disability. The four headache impact severity categories are little or no impact (49 or less), some impact (50–55), substantial impact (56–59), and severe impact (60–78) [24].

Neck pain frequency: This was a self-reported measure, in which the patients were asked to report the number of neck pain days in 4 weeks through a medical log book [22].

Neck pain intensity: The intensity of neck pain was measured on a 10-point visual analogue scale (VAS). Patients were asked to mention their intensity of pain on the 10 cm scale, in which 0 denoted ‘no pain’ and 10 denoted ‘maximum intolerable pain.’ This is a valid and reliable (ICC = 0.60–0.77) tool for measuring neck pain intensity [23].

Neck pain threshold: The threshold of pain was measured with a digital algometer (Wagner, Model FPX, USA). The algometer was applied over the standard locations in the neck region, which were identified by palpation techniques. This is a reliable (intrarater ICC = 0.815–0.940 and test—retest ICC = 0.854–0.906) and valid tool for measuring the pain threshold [25].

Flexion–rotation test (FRT): This was measured using a modified cervical range of motion (CROM) device (Physio supplies, Groningen, Nederland). The device was fixed at the apex of the skull by Velcro straps, and the combined flexion–rotation movement on both sides was measured. The sensitivity and specificity of the FRT in measuring CROM is 90% and 80%, respectively [26].

Neck disability index (NDI): This was a valid and reliable (r = 0.89) self-reporting questionnaire consisting of 10 items measured on a 0- to 5-point scale. The grade of disability of an individual was decided as per the score obtained, such as 0–4: no disability, 5–14: mild disability, 15–24: moderate disability, 25–34: severe disability, and 34 or more: complete disability [27].

Quality of life: The European quality of life five-dimension—EuroQol 5D (mobility, self-care, usual activities, pain/discomfort, and anxiety/depression)—is a psychometric scale that was used to measure the overall quality of life in CGH patients. It had a good test–retest reliability between 0.65 and 0.91, and construct validity was good [28].

### 2.5. Sample Size

The sample size was calculated using a previous pilot study, with primary outcome data of CGH days tested with one-way ANOVA [29]. The required sample size was calculated by assuming 80% power using a two-tailed test with a significance level of 0.05. To detect the minimum effect size of 1.2 CGH days with a mean difference of 3.5 CGH and a standard deviation of 0.7 CGH days, the sample size required was 25 in each group. When considering a 10% dropout, the sample size required in each group was 28.

### 2.6. Randomization 

An individual who was not involved in the data collection was used for randomization. The participants were randomized to MMT, SMT, and control groups with a 1:1:1 allocation ratio. Randomization was performed using computer-generated random allocation cards (RANCODE^®^, IDV, Gauting, Germany). Patients were assigned to one of these 3 groups, and the allocation concealment was done using sealed opaque envelopes. Therefore, the balance between the groups was maintained. All the prospective subjects who fulfilled the eligibility criteria were allowed to participate.

### 2.7. Blinding 

Due to the design and settings of the study, it was not possible to blind the treating therapist and the participants involved in the study. The therapist who assessed the outcomes at baseline, after 4 weeks, after 8 weeks, and at a 6-month follow-up was blinded. Therefore, the treating and assessing therapists were different persons, and the assessing therapist remained blinded. Participants were also instructed not to disclose their study procedures, treatment protocol, and their group allocation with the assessing therapist.

### 2.8. Statistical Methods 

The participants’ demographic and clinical characteristics were presented, tabulated, and analyzed for study homogeneity using the Shapiro–Wilk test. The baseline, 4-week, 8-week, and 6-month follow-up measurements of primary and secondary variables were measured and presented as mean ± standard deviation (SD). The mixed model with repeated measures (MMRM) was performed to measure the group × time effect of all variables. Due to quantitative variables and normal sample distribution, the one-way ANOVA test was performed to find the difference between the treatment groups. Repeated measures analysis of variance (rANOVA) was performed to find the intragroup effects, with planned and corrected Bonferroni post-hoc tests. The 3 × 4 (group × time) repeated measure multivariate analysis of variance (RM-MANOVA), with planned, corrected post-hoc tests for all the outcome variables, were performed between the groups [29]. The statistical analyses were performed using IBM SPSS Statistics for Windows (version 20.0), and the statistically significant value was set at 0.05.

## 3. Results

**Figure 1:**
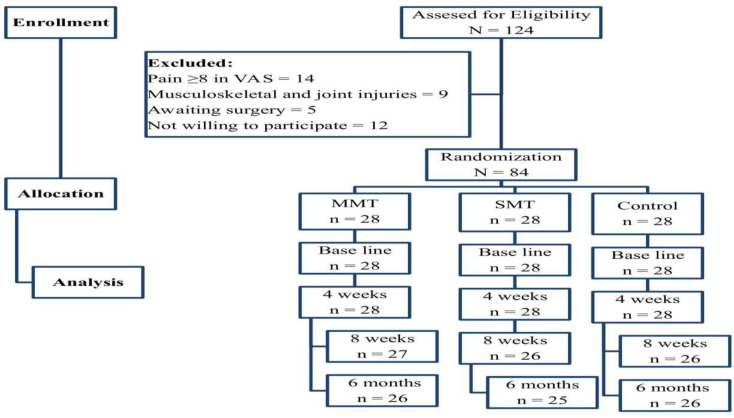
Flow chart showing the study detail.

Out of the 124 participants initially recruited, 14 participants had a pain score more than 8 on the visual analogue scale, 9 had musculoskeletal and joint injuries, 5 were awaiting some sort of surgery, and 12 participants did not consent to participate in the study. Eighty-four (N = 84) participants were eligible to participate, and they were randomized into three groups. Two participants each from the MMT and the control group and three participants from the SMT group dropped out at the end of the 6-month follow-up analysis due to some personal inconveniences (Figure 1). All participants were one-hundred percent compliant with their treatment protocol and did not face any adverse side effects during or after the treatment.

The participants’ demographic and clinical characteristics were analyzed between the groups. The test showed no significant difference in age, height, weight, and body mass index (BMI) measures (*p* > 0.05), and the data were suitable for further statistical analysis. In this study, women (54–57%) were affected more than men in all three groups. The clinical presentation of headache was shown as bilateral form (86–89%) rather than unilateral presentation, and most of the CGH cases had associated neck pain. The clinical variable measures, such as CGH duration, CGH frequency, and CGH intensity, were used to plan the intervention procedure, which showed no significant difference. The data (*p* > 0.05) are presented as mean and SD in Table 1.

**Figure 2:**
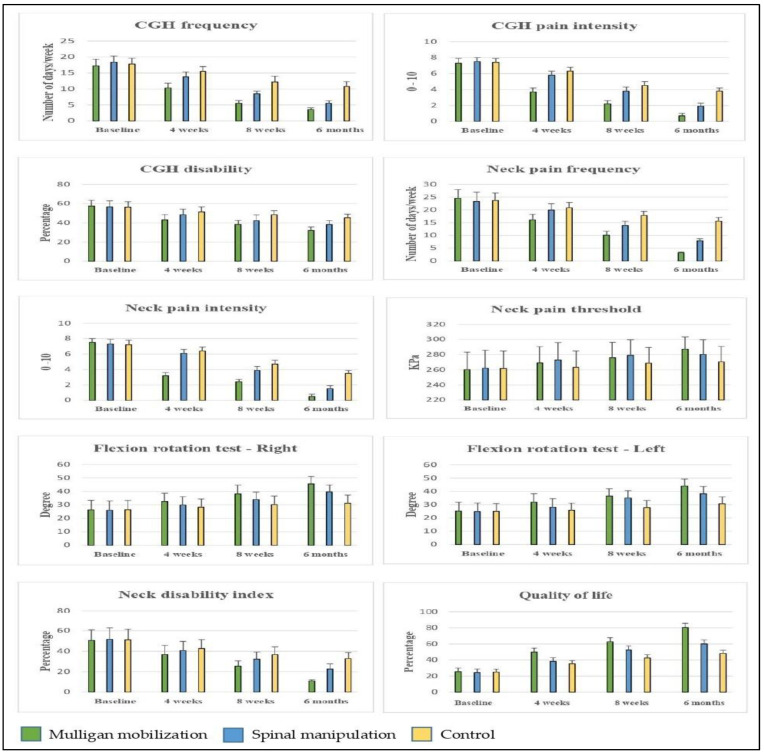
Pre- and post-primary and secondary outcome measure scores of MMT, SMT, and control group.

The baseline primary variable did not show any significant difference (*p* > 0.05) between the MMT, SMT, and control groups. At four weeks of intervention, CGH frequency 5.0 (95% CI 4.04 to 5.95) improved (*p* < 0.001) in the MMT group more than in the SMT and control groups. The same differences were observed at the 8-week and at the 6-month follow-ups. At the end of the 6-month follow-up, the CGH frequency of 7.3 (95% CI 6.63 to 7.96) improved (*p* < 0.001) more in the MMT group than in the SMT group (Table 2 and Table 3). The post-hoc Bonferroni test showed more standard mean difference (SMD) in the primary outcome variable at the 6-month follow-up appointment. On calculating the effect size, the overall changes were noted in the CGH frequency of the MMT group (d = 10.14), SMT group (d = 9.55), and control group (d = 4.24), which was categorized into larger effects. The graphical representation in Figure 2 also shows more improvements in the MMT group than those in the SMT and control groups. At the 6-month follow-up analysis, the minimal clinically important difference score (MCID) in CGH frequency (MCID = 7.3), between the MMT and SMT groups showed better improvement in the MMT group than in the SMT group. The MCID found in CGH frequency was reached in all the groups at the 6-month follow-up. This was reached faster in the MMT group in 4 weeks, but the SMT group reached it at 8 weeks and the control group reached it in the 6-month period.

The secondary outcomes, such as the CGH pain intensity, CGH disability, neck pain frequency, neck pain intensity, neck pain threshold, flexion rotation (right and left), neck disability, and quality of life, were measured. The baseline scores did not show any significant difference (*p* > 0.05) between the groups. After four weeks of intervention, CGH pain intensity 2.6 (95% CI 2.28 to 2.91), CGH disability 8.1 (95% CI 4.67 to 11.66), neck pain frequency 4.7 (95% CI 3.26 to 6.13), neck pain intensity 3.2 (95% CI 2.90 to 3.49), flexion rotation (right) −4.1 (95% CI −8.06 to −0.23), flexion rotation (left) −6.1 (95% CI −9.94 to −2.27), neck disability 5.8 (95% CI 0.17 to 11.4), and quality of life −14.5 (95% CI −17.3 to −11.6) scores improved (*p* < 0.001) more in the MMT group than in the SMT and control groups, but neck pain threshold −5.9 (95% CI −19.85 to 8.05) did not improve more. The same growth was noted in the 8-week and 6-month follow-up. At the end of the 6-month follow-up, CGH pain intensity 3.1 (95% CI 2.86 to 3.33), CGH disability 13.0 (95% CI 10.5 to 15.4), neck pain frequency 12.4 (95% CI 11.7 to 13.0), neck pain intensity 3.0 (95% CI 2.76 to 3.23), neck pain threshold −16.4 (95% CI −28.29 to −4.5), flexion rotation (right) −14.2 (95% CI −17.9 to −10.6), flexion rotation (left) −13.3 (95% CI −16.7 to −9.8), neck disability 21.8 (95% CI 18.64 to 24.99), and quality of life −32.1 (95% CI −35.1 to −29.0) scores showed more improvement (*p* < 0.001) in the MMT group than those in the SMT and control groups (Table 2 and Table 3). The graphical representation in Figure 2 also shows more changes in the MMT group than those in the SMT and control groups.

## 4. Discussion 

This is the first novel randomized, controlled study with the objective of finding and comparing the effects of Mulligan’s mobilization, spinal manipulation, and conventional massage in the management of cervicogenic headache. We observed that the cervical region is the most unstable area in the body and is more prone to joint dysfunctions. Consequently, cervical dysfunctions may result in cervicogenic headache, neck pain, reduced cervical range of motion, and disturbed functional activities [21,30].

The results of the study show that, after four weeks of intervention, primary (CGH frequency) and secondary (CGH pain intensity, CGH disability, neck pain frequency, neck pain intensity, flexion rotation (right and left), neck disability, and quality of life) measures improved more (*p* < 0.001) in the MMT group than those in the SMT and control group, but the neck pain threshold measurement did not improve. The same differences were observed at the 8-week and at the 6-month follow-ups. This study found that four weeks of Mulligan’s manual therapy had a significant effect on both the primary and secondary variables in cervicogenic headache. Mulligan’s manual mobilization technique, such as SNAG, plays an effective role in treating cervicogenic headache and associated neck pain. A recent study reported that such type of mobilization was also helpful in treating tension-induced headache and reduced the frequency of headache, which is also a finding of our study [31]. Manack et al. stated that performing SNAG on the painful cervical vertebra could lead to a reduction in the pain intensity in CGH patients [32]. 

According to Mulligan’s mobilization, the SNAG approach is given to the facet joint till the end range of joint motion, in which a considerable amount of power and direction is maintained. This approach retains the original position of the facet joint of the cervical vertebra and thereby reduces neck dysfunction and improves neck disability. It was also found that biomechanical changes in the vertebra due to MMT affect the central processing and inhibit the pain mechanism [15]. Furthermore, joint mobilization can be helpful in reducing joint adhesions and increasing the pain pressure threshold of the paravertebral muscles. However, our study found that there was a delay in the improvement of the pain pressure threshold at the sensitive points of the paravertebral region. The reports of our study are contradicted by Martínez et al., who pointed out that SMT was superior to different types of mobilization [33]. 

The results of this study also showed improvement in primary and secondary outcomes after spinal manipulation therapy in CGH patients. SMT showed significant improvement in pain intensity and joint mobility, which was supported by a previous study by Giles and Muller et al. [34]. A study by Cassidy et al. stated that SMT is more effective than mobilization technique in reducing pain in cervicogenic headache [35]. Hoving et al. suggested that spinal manipulation accelerated the recovery process of CGH [36]. Despite numerous studies done to find the effects of SMT, the exact biomechanical and neurophysiological mechanisms behind its effects have still not been identified. According to Fernández de Las Peñas et al., SMT inhibits the action of the nociceptive fibers in the facet joints, discs, paravertebral muscles, and soft tissues, which leads to reduced pain and improved joint range. The high-velocity thrust maneuver induces the activity of joint receptors and inhibits the pain pathway through the pain gate mechanism. The biomechanical changes have a significant contribution to sensory receptors such as muscle spindles and Golgi tendon organs, which relaxes the muscles and reduces muscle soreness [37]. 

The minimal effects of conventional massage on pain intensity have been explained by previous studies, where the application of massage induced neurophysiological activity [21]. It activates the opioid and oxytocin interaction and promotes an anti-nociceptive reaction. The other important mechanism of massage on CGH is the desensitization of the trigeminocervical nucleus, which has a considerable role in reducing the tone of the suboccipital and neck muscles [38]. All the participants in the three groups performed the neck isometric exercises, and the exercises were tracked in an exercise log book and supervised regularly by a treating therapist. Therefore, these exercises would not potentially confound the outcome of the results.

The additional beneficial effect of Mulligan mobilization as observed in our study could be due to its ability to reduce joint adhesions and increase the pain pressure threshold when compared with that of spinal manipulation and conventional massage therapy. The reports of this study will be helpful to physical therapists for selecting a proper evidence-based treatment approach for CGH patients. To the best of our knowledge, this is the first study that analyzed the clinical symptoms of CGH and its associated neck problems after different training protocols. On a long-term basis, proper patient education and regular physical exercise would be helpful to maintain and prevent the further progression of the disease. 

The study has certain limitations. First, although the sample size was calculated through the power analysis method, the authors felt the sample size was small and was a potential for type 2 error. Second, both genders were included in the study, but since the data were not analyzed separately during data interpretation, these gender differences might have an impact on the study outcomes. Additionally, due to the nature of the intervention, it was not possible to blind the therapist who administered the intervention to the participants. Third, there is a lack of a placebo or sham group in this study to identify the real effects of treatment groups. A strict physiotherapy protocol prohibited an individual adjustment of treatment and may have influenced the results. Fourth, the duration of the application of different mobilization approaches was not similar, which may affect the outcome of the results. It was also not possible to guarantee that the participants filled out the medical log book every day rather than after a week or four weeks. Finally, to allow future sample size and power calculations based on the presented results, the variance and covariance matrix can be provided, or the correlation between the variables can be presented. In addition, further research is recommended to find the physical and molecular mechanism behind the clinical and physiological effects of these approaches in CGH patients. 

## 5. Conclusions

The study was conducted according to the strict guidelines of CONSORT and concluded that Mulligan’s mobilization with the SNAG approach provided better outcomes in cervicogenic headache than those of spinal manipulation therapy and conventional massage therapy. This study provided sound evidence for the treatment of a widespread and costly clinical condition, namely, cervicogenic headache. Additionally, this study provided a better understanding of this condition and gave clinical evidence for selecting the proper manual therapy technique for cervicogenic headache conditions.

## Figures and Tables

**Table 1:** Demographic details of the MMT, SMT, and control groups.

Sr. No	Variable		MMT	SMT	Control	*p*-Value
1	Age (year)	-	32.8 ± 2.8	33.1 ± 2.6	32.5 ± 2.7	0.709 *
2	Sex	Male	13 (46%)	12 (43%)	13 (46%)	-
		Female	15 (54%)	16 (57%)	15 (54%)	-
3	Height (cm)	-	163.7 ± 4.1	164.5 ± 3.9	163.2 ± 4.3	0.492 *
4	Weight (kg)	-	68.74 ± 3.1	69.88 ± 3.4	69.12 ± 3.5	0.433 *
5	BMI (kg/m^2^)	-	24.2 ± 2.18	23.9 ± 2.23	24.3 ± 1.98	0.766 *
6	CGH duration (year)	-	6.5 ± 3.1	6.8 ± 3.4	5.8 ± 2.9	0.476 *
7	CGH frequency (per day)	-	0.72 ± 0.16	0.68 ± 0.15	0.74 ± 0.13	0.304 *
8	CGH intensity (0–10)	-	6.5 ± 1.6	7.1 ± 1.5	6.8 ± 1.5	0.347 *
9	Headache (side)	Unilateral	24 (86%)	25 (89%)	24 (86%)	-
		Bilateral	4 (14%)	3 (11%)	4 (14%)	-
10	Neck pain	Yes	14	15 (100%)	14	-
	(Associated pain)	No	1	0 (0%)	1	-

* Non-significant, MMT: Mulligan manual therapy, SMT: Spinal manipulative therapy, BMI: Basal metabolic index, CGH: Cervicogenic headache.

**Table 2:** Primary and secondary outcome variable analysis of MMT, SMT, and control groups.

	Variable		MMT	SMT	Control	*p*-Value
1	CGH frequency (no. of days per 4 weeks)	Baseline	17.2 ± 2.1	18.4 ± 1.9	17.8 ± 1.8	0.074 *
		4 weeks	10.3 ± 1.5	13.8 ± 1.5	15.5 ± 1.5	0.001
		8 weeks	5.5 ± 0.9	8.5 ± 0.8	12.2 ± 1.8	0.001
		6 months	3.5 ± 0.6	5.5 ± 0.8	10.8 ± 1.5	0.001
		*p*-value	0.001	0.001	0.001	
2	CGH pain intensity (0–10)	Baseline	7.3 ± 0.6	7.5 ± 0.5	7.4 ± 0.5	0.380 *
		4 weeks	3.7 ± 0.5	5.8 ± 0.5	6.3 ± 0.5	0.000
		8 weeks	2.2 ± 0.4	3.8 ± 0.5	4.5 ± 0.5	0.000
		6 months	0.7 ± 0.3	1.9 ± 0.4	3.8 ± 0.4	0.000
		*p*-value	0.001	0.001	0.001	
3	CGH disability (percentage)	Baseline	57.33 ± 6.2	56.47 ± 6.5	56.22 ± 5.8	0.781 *
		4 weeks	43.11 ± 5.4	48.32 ± 5.8	51.28 ± 5.2	0.001
		8 weeks	38.41 ± 4.1	42.35 ± 5.8	48.37 ± 4.2	0.001
		6 months	32.21 ± 3.6	38.32 ± 4.1	45.22 ± 3.8	0.001
		*p*-value	0.001	0.001	0.001	
4	Neck pain frequency (no. of days per 4 weeks)	Baseline	24.5 ± 3.5	23.3 ± 3.6	23.7 ± 2.9	0.397 *
		4 weeks	16.1 ± 2.1	19.9 ± 2.5	20.8 ± 2.1	0.001
		8 weeks	10.1 ± 1.5	13.9 ± 1.7	17.8 ± 1.6	0.001
		6 months	3.2 ± 0.3	7.9 ± 0.8	15.6 ± 1.4	0.001
		*p*-value	0.001	0.001	0.001	
5	Neck pain intensity (0–10)	Baseline	7.5 ± 0.5	7.3 ± 0.6	7.2 ± 0.6	0.139 *
		4 weeks	3.2 ± 0.4	6.1 ± 0.5	6.4 ± 0.5	0.001
		8 weeks	2.4 ± 0.3	3.9 ± 0.5	4.7 ± 0.5	0.001
		6 months	0.5 ± 0.3	1.5 ± 0.4	3.5 ± 0.4	0.001
		*p*-value	0.001	0.001	0.001	
6	Neck pain threshold (KPa)	Baseline	260.3 ± 22.9	262.2 ± 23.5	261.8 ± 22.9	0.948 *
		4 weeks	269.2 ± 21.3	273.1 ± 22.8	263.3 ± 21.5	0.244 *
		8 weeks	276.1 ± 20.1	278.9 ± 20.5	268.8 ± 20.6	0.167 *
		6 months	287.1 ± 16.2	280.1 ± 19.4	270.7 ± 20.1	0.006
		*p*-value	0.001	0.009	0.341 *	
7	Flexion rotation test (right side—degree)	Baseline	26.22 ± 7.1	25.97 ± 6.8	26.32 ± 6.9	0.981 *
		4 weeks	32.43 ± 6.2	29.88 ± 6.1	28.28 ± 6.1	0.043
		8 weeks	38.17 ± 6.5	33.92 ± 5.5	30.18 ± 6.3	0.001
		6 months	45.48 ± 5.6	39.58 ± 5.2	31.19 ± 6.1	0.001
		*p*-value	0.001	0.001	0.026	
8	Flexion rotation test (left side—degree)	Baseline	25.21 ± 6.6	24.82 ± 6.5	24.92 ± 5.9	0.205 *
		4 weeks	31.92 ± 6.3	28.19 ± 6.3	25.81 ± 5.4	0.001
		8 weeks	36.51 ± 5.5	34.97 ± 5.5	27.82 ± 5.4	0.001
		6 months	43.94 ± 5.3	38.21 ± 5.4	30.62 ± 5.2	0.001
		*p*-value	0.001	0.001	0.001	
9	Neck disability index	Baseline	50.62 ± 10.2	51.58 ± 11.3	51.12 ± 10.3	0.888 *
		4 weeks	36.79 ± 9.0	40.65 ± 8.9	42.62 ± 8.7	0.049
		8 weeks	25.32 ± 5.3	32.15 ± 7.2	36.72 ± 7.5	0.001
		6 months	10.81 ± 1.3	22.56 ± 5.2	32.63 ± 6.2	0.001
		*p*-value	0.001	0.001	0.001	
10	Quality of life	Baseline	25.4 ± 4.7	24.2 ± 4.6	24.9 ± 3.8	0.591 *
		4 weeks	49.8 ± 4.9	38.3 ± 4.7	35.3 ± 3.9	0.001
		8 weeks	62.7 ± 5.1	52.6 ± 4.7	42.6 ± 4.0	0.001
		6 months	80.3 ± 5.4	60.1 ± 4.9	48.2 ± 4.1	0.001
		*p*-value	0.001	0.001	0.001	

* Non-significant, CGH: Cervicogenic headache, MMT: Mulligan manual therapy, SMT: Spinal manipulative therapy.

**Table 3:** Pre- and post-mean difference and confidence interval (upper limit and lower limit) scores of MMT, SMT, and control groups.

Variable/Time		Baseline	4 Weeks	8 Weeks	6 Months
		Mean Difference CI 95% (Upper Limit–Lower Limit)			
CGH frequency					
1	MMT × SMT	1.2 (−0.03 to 2.43)	3.3 (2.34 to 4.25)	3.0 (2.20 to 3.79)	2.0 (1.33 to 2.66)
	MMT × Control	0.6 (−0.60 to 1.83)	5.0 (4.04 to 5.95)	6.7 (5.90 to 7.49)	7.3 (6.63 to 7.96)
	SMT × Control	−0.6 (−1.83 to 0.63)	1.7 (0.74 to 2.65)	3.7 (2.90 to 4.49)	5.3 (4.63 to 5.96)
CGH pain intensity					
2	MMT × SMT	0.2 (−0.14 to 0.54)	2.1 (1.78 to 2.41)	1.6 (1.30 to 1.89)	1.2 (0.96 to 1.43)
	MMT × Control	0.1 (−0.24 to 0.44)	2.6 (2.28 to 2.91)	2.3 (2.00 to 2.59)	3.1 (2.86 to 3.33)
	SMT × Control	−0.1 (−0.44 to 0.24)	0.5 (0.18 to 0.81)	0.7 (0.40 to 0.99)	1.9 (1.66 to 2.13)
CGH disability					
3	MMT × SMT	−0.8 (−4.79 to 3.07)	5.2 (1.71 to 8.70)	3.9 (0.90 to 6.98)	6.1 (3.66 to 8.55)
	MMT × Control	−1.1 (−5.04 to 2.82)	8.1 (4.67 to 11.66)	9.9 (6.92 to 13.0)	13.0 (10.5 to 15.4)
	SMT × Control	−0.2 (−4.18 to 3.68)	2.9 (−0.53 to 6.45)	3.8 (2.77 to 4.82)	6.9 (4.45 to 9.34)
Neck pain frequency					
4	MMT × SMT	−1.2 (−3.33 to 0.93)	3.8 (2.36 to 5.23)	7.7 (6.67 to 8.72)	4.7 (4.07 to 5.32)
	MMT × Control	−0.8 (−2.93 to 1.33)	4.7 (3.26 to 6.13)	3.9 (2.87 to 4.92)	12.4 (11.7 to 13.0)
	SMT × Control	0.4 (−1.73 to 2.53)	0.9 (−0.53 to 2.33)	3.7 (3.54 to 3.85)	7.7 (7.07 to 8.32)
Neck pain intensity					
5	MMT × SMT	−0.2 (−0.56 to 0.16)	2.9 (2.60 to 3.19)	1.5 (1.21 to 1.78)	1.0 (0.76 to 1.23)
	MMT × Control	−0.3 (−0.66 to 0.06)	3.2 (2.90 to 3.49)	2.3 (2.01 to 2.58)	3.0 (2.76 to 3.23)
	SMT × Control	−0.1 (−0.46 to 0.26)	0.3 (0.00 to 0.59)	0.8 (0.51 to 1.00)	2.0 (1.76 to 2.23)
Neck pain threshold					
6	MMT × SMT	1.9 (−12.84 to 16.64)	3.9 (−10.05 to 17.85)	2.8 (−10.21 to 15.81)	−7.0 (−18.8 to 4.89)
	MMT × Control	1.5 (−13.24 to 16.24)	−5.9 (−19.85 to 8.05)	−7.3 (−20.31 to 5.71)	−16.4 (−28.2 to −4.5)
	SMT × Control	−0.4 (−15.14 to 14.34)	−9.8 (−23.75 to 4.15)	−10.1 (−23.11 to 2.9)	−9.4 (−21.2 to 2.49)
Flexion rotation test (right side)					
7	MMT × SMT	−0.2 (−4.67 to 4.17)	−2.5 (−6.46 to 1.36)	−4.2 (−7.83 to −0.66)	−5.9 (−9.58 to −2.21)
	MMT × Control	−0.2 (−4.67 to 4.17)	−4.1 (−8.06 to −0.23)	−7.9 (−11.57 to −4.4)	−14.2 (−17.9 to −10.6)
	SMT × Control	0.3 (−4.07 to 4.77)	−1.6 (−5.51 to 2.31)	−3.7 (−7.32 to −0.15)	−8.3 (−2.0 to −4.7)
Flexion rotation test (left side)					
8	MMT × SMT	−0.3 (−4.43 to 3.65)	−3.7 (−7.56 to 0.10)	−1.5 (−5.02 to 1.94)	−5.7 (−9.15 to −2.3)
	MMT × Control	−0.2 (−4.33 to 3.75)	−6.1 (−9.94 to −2.27)	−8.6 (−12.17 to −5.2)	−13.3 (−16.7 to −9.8)
	SMT × Control	0.1 (−3.94 to 4.14)	−2.38 (−6.21 to 1.45)	−7.1 (−10.63 to −3.66)	−7.5 (−11.0 to −4.16)
Neck disability index					
9	MMT × SMT	0.9 (−5.81 to 7.73)	3.8 (−1.79 to 9.51)	6.8 (2.53 to 11.12)	11.7 (8.57 to 14.9)
	MMT × Control	0.5 (−6.27 to 7.27)	5.8 (0.17 to 11.4)	11.4 (7.10 to 15.69)	21.8 (18.64 to 24.99)
	SMT × Control	−0.4 (−7.23 to 6.31)	1.9 (−3.68 to 7.62)	4.5 (0.27 to 8.86)	10.0 (6.89 to 13.2)
Quality of life					
10	MMT × SMT	−1.2 (−3.99 to 1.59)	−11.5 (−14.38 to −8.6)	−10.1 (−13.0 to −7.1)	−20.2 (−23.2 to −17.1)
	MMT × Control	−0.5 (−3.29 to 2.29)	−14.5 (−17.3 to −11.6)	−20.1 (−23.0 to −17.1)	−32.1 (−35.1 to −29.0)
	SMT × Control	0.7 (−2.09 to 3.49)	−3.0 (−5.88 to −0.11)	−10.0 (−12.9 to −7.05)	−11.9 (−14.9 to −8.81)

MMT: Mulligan manual therapy, SMT: Spinal manipulative therapy.

## Data Availability

Data are not publicly available but can be obtained from the corresponding author on request.

## References

[1] Boardman H., Thomas E., Croft P., Millson D. (2003). Epidemiology of Headache in an English District. Cephalalgia.

[2] Strimpakos N. (2011). The assessment of the cervical spine. Part 1, Range of motion and proprioception. J. Bodyw. Mov. Ther..

[3] Headache Classification Committee of the International Headache Society (IHS) (2013). The International Classification of Headache Disorders, 3rd edition (beta version). Cephalalgia.

[4] Haldeman S., Dagenais S. (2001). Cervicogenic headaches: A critical review. Spine J..

[5] Atlas of Headache Disorders and Resources in the World 2011 (2011). A Collaborative Project of the World Health Organization and Lifting the Burden.

[6] Solomon G.D., Cady R.K., Klapper J.A., Ryan R.E. (1997). Standards of care for treating headache in primary care practice. National Headache Foundation. Cleve Clin. J. Med..

[7] Classification Committee of the International Headache Society (2004). The International Classification of Headache Disorders: 2nd edition. Cephalalgia.

[8] Zito G., Jull G., Story I. (2006). Clinical tests of musculoskeletal dysfunction in the diagnosis of cervicogenic headache. Man. Ther..

[9] Hall T.M., Briffa K., Hopper D., Robinson K. (2010). Comparative analysis and diagnostic accuracy of the cervical flexion–rotation test. J. Headache Pain.

[10] Takasaki H., Hall T., Oshiro S., Kaneko S., Ikemoto Y., Jull G. (2011). Normal kinematics of the upper cervical spine during the Flexion–Rotation Test—In vivo measurements using magnetic resonance imaging. Man. Ther..

[11] Kristoffersen E.S., Lundqvist C. (2014). Medication-overuse headache: A review. J. Pain Res..

[12] Hudson J.S., Ryan C.G. (2010). Multimodal group rehabilitation compared to usual care for patients with chronic neck pain: A pilot study. Man. Ther..

[13] Clarke T.C., Black L.I., Stussman B.J., Barnes P.M., Nahin R.L. (2015). Trends in the use of complementary health approaches among adults: United States, 2002–2012. Natl. Health Stat. Rep..

[14] Cleland J.A., Childs M.J.D., McRae M., Palmer J.A., Stowell T. (2005). Immediate effects of thoracic manipulation in patients with neck pain: A randomized clinical trial. Man. Ther..

[15] Mulligan B.R. (2010). Manual Therapy: Nags, Snags, Mwms, Etc..

[16] Bronfort G., Haas M., Evans R., Leininger B., Triano J. (2010). Effectiveness of manual therapies: The UK evidence report. Chiropr. Man. Ther..

[17] Haas M., Bronfort G., Evans R.L. (2006). Chiropractic Clinical Research: Progress and Recommendations. J. Manip. Physiol. Ther..

[18] Bryans R., Descarreaux M., Duranleau M., Marcoux H., Potter B., Ruegg R., Shaw L., Watkin R., White E. (2011). Evidence-Based Guidelines for the Chiropractic Treatment of Adults with Headache. J. Manip. Physiol. Ther..

[19] Munoz-Ceron J., Marin-Careaga V., Peña L., Mutis J., Ortiz G. (2019). Headache at the emergency room: Etiologies, diagnostic usefulness of the ICHD 3 criteria, red and green flags. PLoS ONE.

[20] Peterson D.H., Bergmann T. (2010). Chiropractic Technique: Principles and Procedures.

[21] Hopper D., Bajaj Y., Choi C.K., Jan O., Hall T., Robinson K., Briffa K. (2013). A pilot study to investigate the short-term effects of specific soft tissue massage on upper cervical movement impairment in patients with cervicogenic headache. J. Man. Manip. Ther..

[22] Bendtsen L., Bigal M.E., Cerbo R., Diener H.C., Holroyd K., Lampl C., Mitsikostas D.D., Steiner T.J., Tfelt-Hansen P. (2010). International Headache Society Clinical Trials S: Guidelines for controlled trials of drugs in tension-type headache: Second edition. Cephalalgia.

[23] Jensen M.P., Karoly P., Braver S. (1986). The measurement of clinical pain intensity: A comparison of six methods. Pain.

[24] Kosinski M., Bayliss M.S., Bjorner J.B., Ware J.E., Garber W.H., Batenhorst A., Cady R., Dahlof C.G., Dowson A., Tepper S. (2003). A six-item short-form survey for measuring headache impact: The HIT-6. Qual. Life Res. Int. J. Qual. Life. Asp. Treat Care Rehab..

[25] Chesterton L.S., Sim J., Wright C.C., Foster N.E. (2007). Interrater Reliability of Algometry in Measuring Pressure Pain Thresholds in Healthy Humans, Using Multiple Raters. Clin. J. Pain.

[26] Ogince M., Hall T., Robinson K., Blackmore A.M. (2007). The diagnostic validity of the cervical flexion-rotation test in C1/2-related cervicogenic headache. Man Ther..

[27] Vernon H., Mior S. (1991). The Neck Disability Index: A study of reliability and validity. J. Manip. Physiol. Ther..

[28] Luo N., Johnson J.A., Shaw J.W., Feeny D., Coons S.J. (2005). Self-Reported Health Status of the General Adult U.S. Population as Assessed by the EQ-5D and Health Utilities Index. Med. Care.

[29] Haas M., Spegman A., Peterson D., Aickin M., Vavrek D. (2010). Dose response and efficacy of spinal manipulation for chronic cervicogenic headache: A pilot randomized controlled trial. Spine J..

[30] Panjabi M.M., Cholewicki J., Nibu K., Grauer J., Babat L.B., Dvorak J. (1998). Critical load of the human cervical spine: An in vitro experimental study. Clin. Biomech..

[31] Castien R.F., van der Windt D.A., Blankenstein A.H., Heymans M.W., Dekker J. (2012). Clinical variables associated with recovery in patients with chronic tension-type headache after treatment with manual therapy. Pain.

[32] Manack A.N., Buse D.C., Lipton R.B. (2011). Chronic Migraine: Epidemiology and Disease Burden. Curr. Pain Headache Rep..

[33] Martínez-Segura R., Fernández-De-Las-Peñas C., Ruiz-Sáez M., López-Jiménez C., Rodríguez-Blanco C. (2006). Immediate Effects on Neck Pain and Active Range of Motion after a Single Cervical High-Velocity Low-Amplitude Manipulation in Subjects Presenting with Mechanical Neck Pain: A Randomized Controlled Trial. J. Manip. Physiol. Ther..

[34] Giles L.G.F., Muller R. (2003). Chronic spinal pain: A randomized clinical trial comparing medication, acupuncture, and spinal manipulation. Spine.

[35] Cassidy J.D., Lopes A.A., Yong-Hing K. (1992). The immediate effect of manipulation versus mobilization on pain and range of motion in the cervical spine: A randomized controlled trial. J. Manip. Physiol. Ther..

[36] Hoving J.L., Koes B.W., de Vet H.C., van der Windt D.A., Assendelft W.J., van Mameren H., Bouter L.M. (2002). Manual therapy, physical therapy, or continued care by a general practitioner for patients with neck pain. A randomized, controlled trial. Ann. Intern Med..

[37] de las Peñas C.F., Downey C., Page J.M. (2005). Immediate changes in radiographically determined lateral flexion range of motion following a single cervical HVLA manipulation in patients presenting with mechanical neck pain: A case series. Int. J. Osteopath. Med..

[38] Panneton W.M., Gan Q., Livergood R.S. (2011). A Trigeminoreticular Pathway: Implications in Pain. PLoS ONE.

